# Targeting the Subthalamic Nucleus in MEGDEL Syndrome: A Case Report of a Clinically Effective Intervention for Debilitating Lingual Dyskinesia and Dystonia

**DOI:** 10.1002/mdc3.70829

**Published:** 2026-09-30

**Authors:** Philipp Köberle, Florian Lange, Ghadir Abbas, Philipp Capetian, Marius Vogt, Patrick Fricke, Cordula Matthies, Jens Volkmann, Martin Reich, Thomas Musacchio

**Affiliations:** ^1^ Universitatsklinikum Würzburg Neurologische Klinik und Poliklinik Würzburg Germany; ^2^ Institut für Diagnostische und Interventionelle Neuroradiologie Würzburg Germany; ^3^ Neurochirurgische Universitätsklinik Würzburg Würzburg Germany

**Keywords:** MEGDEL syndrome, subthalamic nucleus, lingual dyskinesia, dystonia, deep brain stimulation (DBS)

Mitochondrial diseases exhibit heterogeneous pathophysiologies and phenotypes that, depending on severity, may substantially impair health, development, and life quality. This heterogeneity makes accurate disease‐specific treatment indispensable. This applies in particular to MEGDEL syndrome (associated with mutations in SERAC1): in the largest published cohort (n = 67), 58% of the patients exhibited oropharyngeal dyskinesia and tongue protrusion, whereas dystonic symptoms were observed in 82%.^1^ Whereas a prior report documents successful deep brain stimulation (DBS) of the globus pallidus internus (GPi) in MEGDEL syndrome (Sharma et al. 2021),^2^ this report details the first therapeutically successful intervention targeting the subthalamic nucleus (STN).

## Case Report

Our patient, born in July 2007, developed postnatal respiratory disorder and severe metabolic decompensation, with progressive lactic acidosis and hyperammonemia. Neuropediatric examination and cranial magnetic resonance imaging (MRI) were unremarkable. Urinary analysis showed elevated 3‐methylglutaconic and 3‐methylglutaric acids, suggesting a mitochondrial disorder. This suspicion was confirmed by whole‐exome sequencing in April 2014, which identified a pathogenic SERAC1 variant, establishing the diagnosis of MEGDEL syndrome.

During childhood, the patient exhibited global developmental delay across cognitive, language, and motor domains, with attention deficits, limited vocabulary, and motor fatigability. Progressive neurological deterioration ensued, characterized by slowing of movements, axial and proximal weakness, hypotonia, impaired postural control, gait abnormalities, and reduced endurance. An MRI performed in January 2022 demonstrated bilateral T2‐weighted hyperintensities, with associated atrophy predominantly affecting the rostral putamen, caudate nucleus, globus pallidus, and periaqueductal region consistent with prior reports (Wortmann et al., 2015)^3^ (Fig. 1A,B).

**FIG. 1:**
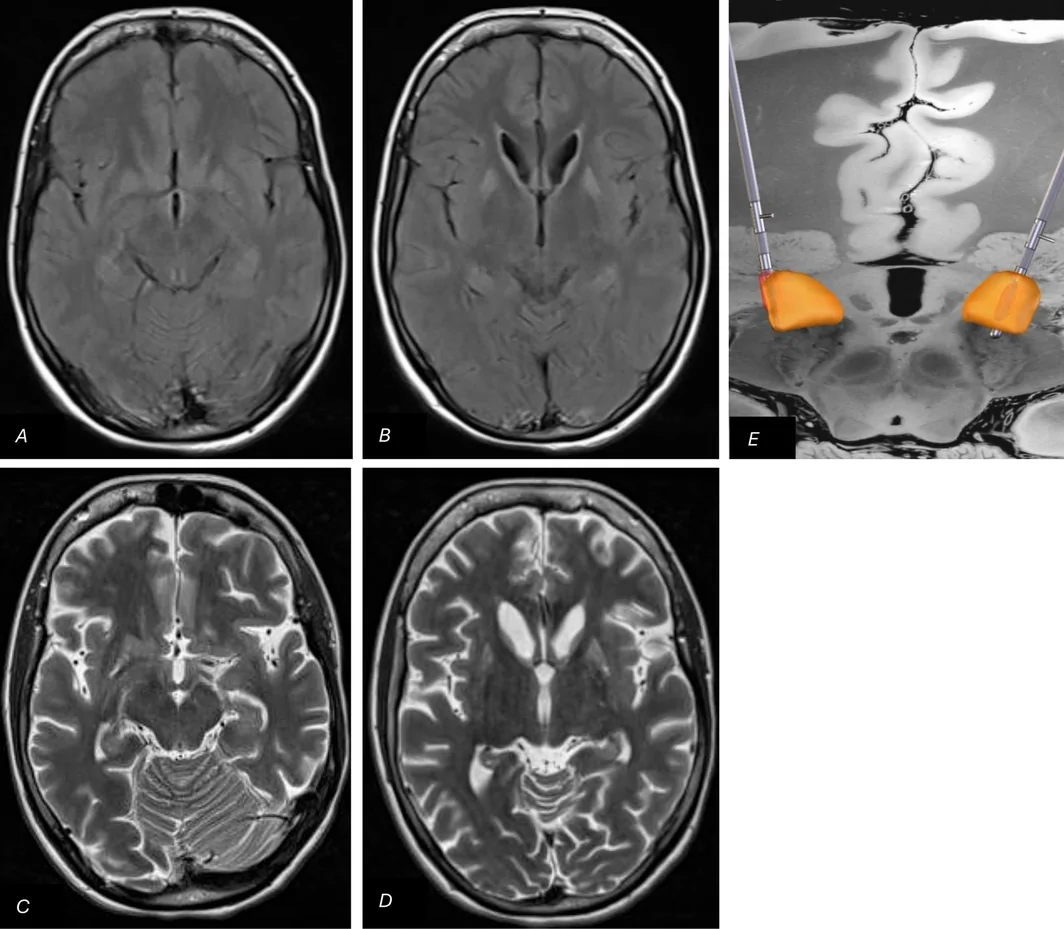
**(A)** MRI (magnetic resonance imaging) FLAIR (fluid‐attenuated inversion recovery) axial 2 mm, 2022; level STN (subthalamic nucleus). **(B)** MRI FLAIR axial 2 mm, 2022; level GPi (globus pallidus internus): T2‐signal hyperintensities at rostral putamen, caudate nucleus, globus pallidus, and periaqueductal region. **(C)** MRI T2 TSE (turbo spin echo) axial 2 mm, 2025; level STN. **(D)** MRI T2 TSE axial 2 mm, 2025; level GPi: progression of T2‐signal hyperintensities. **(E)** Reconstruction of DBS (deep brain stimulation) electrodes (Lead‐DBS/postmortem 7 T; STN orange, volume tissue activated red). In addition to the stimulation of the STN, the stimulation field extends dorsally, engaging pallidofugal fibers that may contribute to the clinical response.

From 2023 onward, the patient developed a severe oromandibular movement disorder dominated by progressive lingual dystonic dyskinesia with involuntary tongue protrusion and caudal flexion, resulting in impaired speech, reduced oral intake, and chronic inflammatory tongue changes (Fig. 2A,B).

**FIG. 2:**
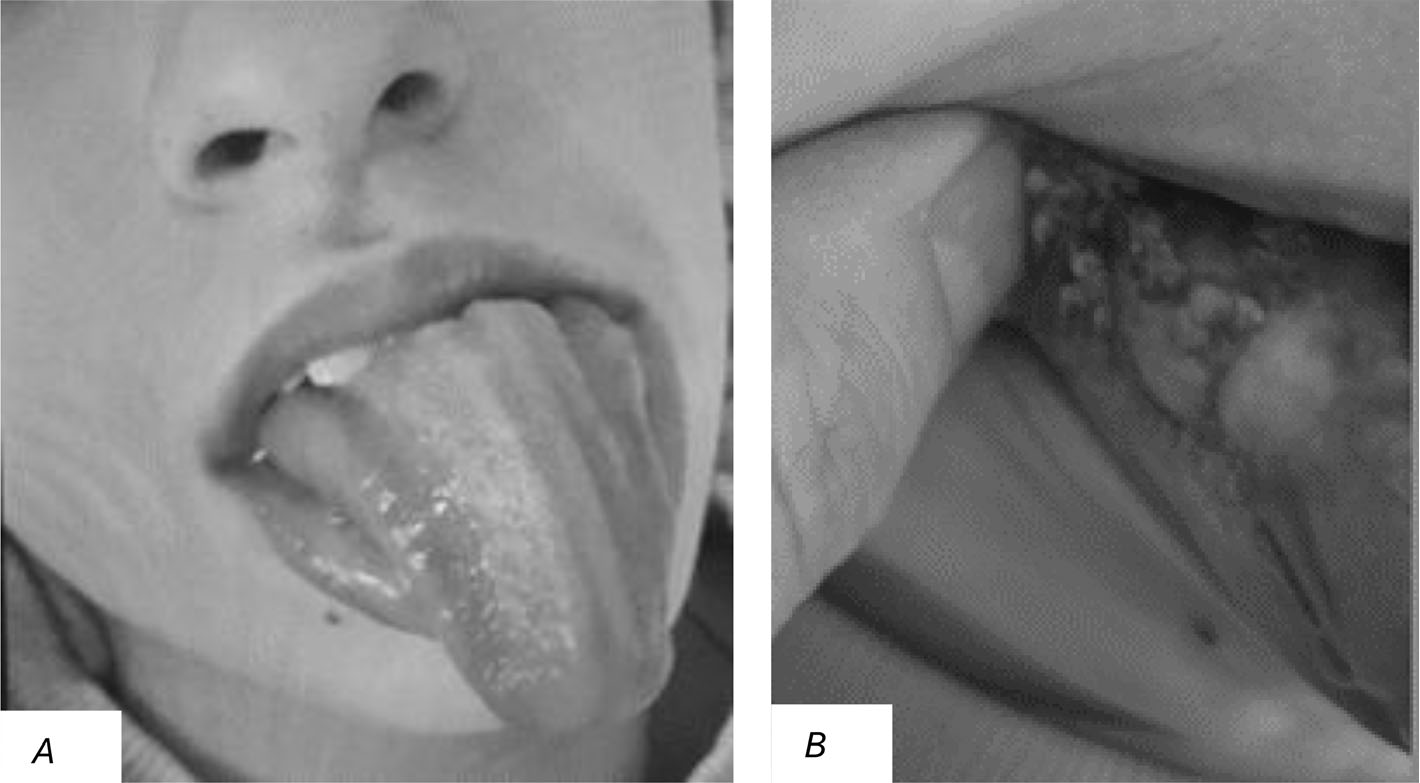
**(A)** Dystonic tongue protrusion without voluntary activation prior to deep brain stimulation. **(B)** Chronic and partially inflammatory changes on the right lateral edge of the tongue as a result of tongue dystonia (2024).

Dysphagia, associated with pseudohypersalivation, resulted in significant underweight (body mass index [BMI]: 16.1 kg/m^2^). Neurological examination recorded symmetrically brisk deep tendon reflexes, generalized muscle atrophy, intermittent painful dystonic muscle spasms, and thoracic dextroconvex spinal malalignment. No further dystonic symptoms were described. Additionally, the patient exhibited a generalized slowing of movements, which in part fulfilled the criteria of bradykinesia (Video 1; Appendix S1).

**Video 1:** Neurological presentation 02/25, before STN‐DBS (subthalamic nucleus deep brain stimulation).

Pseudohypersalivation was treated with botulinumtoxin. A percutaneous endoscopic gastrostomy was placed in May 2024.

A follow‐up brain MRI in February 2025 confirmed bilateral T2‐weighted hyperintensities involving the rostral putamen, the pallidum, and the periaquaeductal gray matter along with atrophy of the caudate nucleus. The pathognomonic putaminal eye sign was not visible. In susceptibility‐weighted imaging nucleus ruber and substantia nigra appeared slightly hypointense (Fig. 1C,D).

In a multidisciplinary conference (neurosurgery/neurology) in March 2025, the STN was determined as the target for DBS implantation. This was due to the structural alterations of the GPi and emerging evidence supporting the STN as an effective target of dystonia, particularly with regard to orofacial symptoms (Butenko et al., 2024),^4^ (Ostrem et al., 2017),^5^ (Wang et al., 2025).^6^ The concomitant slowness of movements supported the choice.

Stereotactic implantation, including microelectrode recording (MER) and intraoperative test stimulation of bilateral multidirectional DBS electrodes (Boston Scientific Cartesia) into the subthalamic nucleus, was performed subsequently on April 23, 2025 (Stimulator: Boston Scientific Vercise Genus R16). The MER showed a well‐preserved, STN‐typical signal along the chosen trajectory on both hemispheres. DBS was activated 3 days after implantation, after image fusion of postoperative CT and preoperative MRI (Fig. 1E; Appendix S2).

The lingual movement disorder improved after DBS activation, with a gradual response developing within 48 hours of initiating STN stimulation. No microlesion effect was evident. An intermittent dyskinesia of the right hand occurred, which again resolved the following week spontaneously.

Three‐month clinical follow‐up (Appendix S3): examination revealed a pronounced improvement in lingual dystonia in the sense that the tongue could again be fully retracted into the mouth. A complete resolution of dystonic muscle spasms was also reported. Fast ambulation and even running were feasible. Oral intake in small quantities became possible, and the patient gained 5 kg of body weight. Bilateral stimulation amplitude was increased by 0.3 mA, inducing appendicular dyskinesia, and was subsequently reverted.

Six‐month clinical follow‐up (Video 2): symptom control remained stable. The parents reported substantial improvements in quality of life and sleep. No clinically relevant stimulation‐related adverse effects were observed. No adjustments of DBS were conducted.

**Video 2:** Neurological presentation 10/25, 6‐month clinical follow‐up after STN‐DBS (subthalamic nucleus deep brain stimulation).

Ten‐month clinical follow‐up: the possibility of daily oral intake further improved, and another 4 kg of weight gain was accomplished, reaching 31 kg at a height of 157 cm (BMI: 16.6 kg/m^2^). The patient regained the ability to speak individual words. The walking distance increased to 300 m. No adjustments were made to DBS.

Fourteen‐month clinical follow‐up: symptom control remained stable. No clinically relevant stimulation‐related adverse effects were observed. No adjustments were made to DBS.

## Discussion

MEGDEL syndrome, a rare mitochondrial disease caused by pathogenic SERAC1 variants, leads to progressive neurodevelopmental impairment and movement disorder. We report the first therapeutically successful STN‐DBS in a patient diagnosed with MEGDEL syndrome presenting with severe lingual dyskinesia and dystonia. Post‐DBS, these symptoms substantially alleviated, and oral intake increased. Additional functional gains included ambulation and a markedly improved quality of life and sleep. No clinically relevant stimulation‐related adverse effects were evident.

## Author Roles

(1) Research Project: A. Conception, B. Organization, C. Execution; (2) Manuscript Preparation: A. Writing of the First Draft, B. Review and Critique.

P.K.: 1A, 1B, 1C, 2A.

F.L.: 1C, 2B.

G.A.: 1C.

P.C.: 2B.

M.V.: 1C.

P.F.: 2B.

C.M.: 2B.

J.V.: 2B.

M.R.: 1A, 2B.

T.M.: 1A, 1B, 1C, 2B.

## Disclosures

**Ethical Compliance Statement**: A written informed consent for the usage of images and videos was provided by the mother of the patient (designated with the power of attorney). The authors confirm that the approval of an institutional review board was not required for this work. They confirm that they have read the journal's position on issues involved in ethical publication and affirm that this work is consistent with those guidelines.

**Financial Disclosures and Conflicts of Interest:** Author disclosures are available in the Supporting Information.

## Supporting information


**Data S1.** Supporting Information.

**Appendix S1.** Neurological examination 02/25, before STN‐DBS (subthalamic nucleus deep brain stimulation).

**Appendix S2.** Initial stimulation parameters of STN‐DBS (subthalamic nucleus deep brain stimulation) 3 days after implantation.

**Appendix S3.** Neurological examination 07/25, 3 months after STN‐DBS (subthalamic nucleus deep brain stimulation).

## Data Availability

The data that support the findings of this study are available from the corresponding author upon reasonable request.
