# Supplementary material for: Targeting the Subthalamic Nucleus in MEGDEL Syndrome: A Case Report of a Clinically Effective Intervention for Debilitating Lingual Dyskinesia and Dystonia
Source: Mov Disord Clin Pract. 2026 Sep 30:10.1002/mdc3.70829. Online ahead of print. doi: 10.1002/mdc3.70829 (PMC13625148; doi:10.1002/mdc3.70829)
Supplement: Supplementary file 1 [file MDC3-9999-0-s002.pdf]

Appendix 1

The patient was alert. Verbal output was limited to unintelligible vocalizations; however, she could communicate non-verbally with head nods and shakes in response to simple questions. She followed commands, albeit with significant latency. The most prominent finding was a severe lingual dystonia, with the tongue constantly protruded and tonically flexed inferiorly. While some residual voluntary control was present (the ability to briefly retract and wiggle the tongue), it appeared flaccid but was clearly tonic on palpation. Her gait was unassisted but unsteady, characterized by internal hip rotation and slight flexion at the hips and knees. Associated arm swing was markedly reduced, and a significant anterior head tilt was noted. There were no other dystonic stigmata. She was unable to stand on her heels or perform a tandem walk. Motor examination revealed globally reduced strength. Tone in the legs was hypotonic, while spasticity was evident in the arms, more pronounced on the left (Ashworth Grade 2). Deep tendon reflexes were brisk bilaterally, with an expanded reflexogenic zone and exhaustible ankle clonus (3 beats). Trömner's sign was positive bilaterally, while the plantar response was negative. Coordination testing (e.g., finger-to-nose) was markedly slow (bradykinetic) but was metrically accurate and non-ataxic.
