# Supplementary material for: Targeting the Subthalamic Nucleus in MEGDEL Syndrome: A Case Report of a Clinically Effective Intervention for Debilitating Lingual Dyskinesia and Dystonia
Source: Mov Disord Clin Pract. 2026 Sep 30:10.1002/mdc3.70829. Online ahead of print. doi: 10.1002/mdc3.70829 (PMC13625148; doi:10.1002/mdc3.70829)
Supplement: Supplementary file 2 [file MDC3-9999-0-s003.pdf]

**Appendix 3**

On examination, the patient was awake and cooperative, following commands purposefully but with marked slowness (bradykinesia). Orientation could not be formally assessed, but she communicated effectively using thumbs up/down gestures. The lingual dystonia had markedly improved; at rest, the tongue was held in a neutral position just behind the lower teeth and could be moved freely without tonic posturing. Motor examination revealed persistent elevated muscle tone, most prominent in the left arm and right leg. Globally reduced strength was still present. Deep tendon reflexes remained brisk bilaterally with an expanded reflexogenic zone. Ankle clonus was present and exhaustible (4 beats on the right, 3 on the left). The plantar response was negative. Coordination testing (e.g., finger-to-nose) remained slow but was metrically accurate and non-ataxic. Her gait was unassisted but remained unsteady, characterized by internal hip rotation, slight flexion at the hips and knees, and markedly reduced arm swing. She could now initiate a tandem walk and stand on her toes with support.
