# Supplementary material for: Targeting the Subthalamic Nucleus in MEGDEL Syndrome: A Case Report of a Clinically Effective Intervention for Debilitating Lingual Dyskinesia and Dystonia
Source: Mov Disord Clin Pract. 2026 Sep 30:10.1002/mdc3.70829. Online ahead of print. doi: 10.1002/mdc3.70829 (PMC13625148; doi:10.1002/mdc3.70829)
Supplement: Supplementary file 3 [file MDC3-9999-0-s004.pdf]

Appendix 2

Left STN: Contacts plane 2 A/B/C (ring stimulation), plane 3 A/B/C (ring stimulation); Amplitude1,3 mA; Pulse Width 60µs; Frequency 130 Hz

Right STN: Contacts plane 2A/B/C (ring stimulation), 3A/B/C (ring stimulation), Amplitude 1,3 mA, Pulse Width 60 µs; Frequency 130 Hz

Electrodes: Boston Scientific Vercise cartesia; 30cm 8 Contact DBS Directional Lead Kit, Ref M365DB2202300
